# Single fathers sacrifice their broods and re-mate quickly in a socially monogamous cichlid

**DOI:** 10.1093/beheco/arad045

**Published:** 2023-06-20

**Authors:** Holger Zimmermann, Kristina M Sefc, Aneesh P H Bose

**Affiliations:** Institute of Biology, University of Graz, Universitätsplatz 2, 8010 Graz, Austria; Institute of Biology, University of Graz, Universitätsplatz 2, 8010 Graz, Austria; Institute of Biology, University of Graz, Universitätsplatz 2, 8010 Graz, Austria

**Keywords:** infanticide, paternal care, pair-bonding, partner loss, re-mating, *Variabilichromis moorii*

## Abstract

When one of two parents disappears in the midst of caring for offspring, the remaining parent is left with several options. They can either (1) desert the brood, (2) continue caring on their own and reject propositions from new potential partners, or (3) continue caring but remain receptive to re-mating opportunities. The presence of a brood may increase re-mating success of single parents, either because brood care is perceived as a signal of partner quality, or because prospective mates perceive the brood as potential energy source. In this field experiment, we used the socially monogamous, biparental cichlid fish *Variabilichromis moorii* to examine the re-mating strategy of males with or without dependent offspring after the loss of their female partner. Partner vacancies were filled quickly by new females, and these females engaged in high levels of affiliative behavior with the males. The new females engaged in territorial defense, but focused primarily against intruding conspecifics, likely as a means to repel rivals. The males, in turn, took over the majority of territorial defense against intruding heterospecifics. Interestingly, males that still had offspring from their previous partnerships did not show aggression toward their new female partners, even when those females were infanticidal and cannibalizing the males’ current offspring. Overall, our experiment shows that single fathers of a biparental species will re-mate quickly even at the detriment to their current offspring.

## Teaser

When a parent disappears while caring for young, the remaining parent is left with a difficult decision: namely, how to balance continued care for their offspring with obtaining a new reproductive partner. We tested how males of a biparental cichlid react when they experimentally separated from their partner, with or without their offspring. Males accepted new females quickly regardless of offspring presence and even allowed the females to cannibalize the brood.

## INTRODUCTION

When an individual loses their mating partner, it is in their reproductive interests to find a replacement partner quickly. However, when an individual loses their partner in the midst of providing biparental care, the single parent is left with several options. They can either (1) desert the brood, (2) continue caring on their own and reject propositions from new potential partners, or (3) continue caring but remain receptive to re-mating opportunities (see Trivers 1972; Dawkins and Carlisle 1976). Care or abandonment decisions depend on a number of factors (Székely et al. 1996), including the ability of the single parent to complete care on their own, the reproductive value of the current brood, and the trade-off between parental care and their ability to attract a new mate. Another important factor is the behavior of new partners toward young that are not their own. Offspring may help to attract new partners if brood care is perceived as a signal of partner quality. But offspring can also be put at risk by the new partner if, for example, killing and cannibalizing them provides energetic benefits or allows the new partner to mate with the parent sooner (Ebensperger 1998; Palombit 2015). While there is evidence that in some species new partners will care for non-related offspring (Yanagisawa and Ochi 1986; Rohwer et al. 1999), parental care and re-pairing are often at odds with one another because new potential mating partners can be infanticidal (Ebensperger 1998; Palombit 2015).

In numerous taxa, non-parental adults may kill dependent offspring so that the parent can redirect resources to reproduction that the newcomer can participate in (Hrdy 1979). The threat of new mates being infanticidal generates conflicting options for the single parent with variation in fitness consequences across the alternatives. The choice to continue care while rejecting new partners may be optimal if the costs of uniparental care are low and/or if the brood is valuable and should not be risked with a potentially infanticidal new partner. The choice to continue care but to still remain receptive to new partners may be optimal if the costs of uniparental care are low and if the new partner is either tolerant of the offspring or if lost offspring can be replaced quickly. For example, if parental care itself is a sexually selected trait that provides mate attraction benefits (Goldberg et al. 2020) then continuing care, at least until a new partner arrives, can allow quick re-mating regardless of whether the new partner is infanticidal. Lastly, the choice to abandon care may be optimal if the costs of uniparental care are too high.

New potential mating partners may be courted by the single parent, or they may take over vacancies forcefully regardless of the single parents’ re-mating preferences. Takeover events are often associated with new individuals killing dependent offspring, and this has been studied primarily in mammals (Hrdy 1974; Ebensperger 1998; Palombit 2015; Pusey and Packer 2016) and birds (e.g., Veiga 2004), and to a lesser extent in insects (Polis 1984; Turillazzi and Cervo 2016) and fishes (Dominey and Blumer 1984). In many species, males that take over groups containing caregiving females can reduce the time until the females become receptive again by removing their current offspring, and this may sometimes involve aggression toward the mothers when they are unwilling to sacrifice their offspring; this is widely known as the sexual selection hypothesis for infanticide (Hrdy 1979). Such takeover mediated infanticide can also occur in fishes and is nearly always committed via cannibalism, which is a common and widespread behavior among caregiving species (Smith and Reay 1991; Manica 2002; Bose 2022). In the cooperatively breeding cichlid *Neolamprologus pulcher,* foreign males that take over a vacant dominant position in a group will quickly kill and eat any dependent offspring. The mothers, however, show strong aggression toward the newcomer males suggesting that their preference is to continue care rather than re-pair immediately (Jindal et al. 2017). The resistance that single parents show toward propositions or takeovers from alternate mating partners should therefore reflect their re-mating preferences. If their preference is to continue caring for the offspring and delay re-mating, then the parent is expected to show aggression toward new prospective mates, especially if the newcomers are threatening to the offspring. However, if their preference is to re-mate quickly, then the parent is expected to show little or no aggression to the new prospective mate, and may even be passive if the newcomer commits infanticide.

In this field experiment, we used the socially monogamous biparental cichlid *Variabilichromis moorii* to examine the re-mating strategy of males after the experimental removal of their female breeding partner. Males and females pair up size assortative (Karino 1997; Zimmermann et al. 2019) and occupy rocky territories in the shallow littoral zones along the shores of southern Lake Tanganyika, East Africa to feed on algae and raise their offspring (Karino 1998; Sturmbauer, Fuchs, et al. 2008; Sturmbauer, Hahn, et al. 2008; Takeuchi et al. 2010). We examined the behavior of males and their re-mating patterns after partner loss, comparing males that had dependent offspring from their previous partnership with males where the offspring were experimentally removed. We expected males with no female partner and no offspring to be willing to re-mate quickly. However, we expected males with no female partner, but with a brood, to face a dilemma. We evaluated how the males responded to our treatments, recording whether they chose to abandon or stay at their territory, and how they behaved socially with new prospective partners, paying close attention to aggression, affiliation, their division of territory defense, and the behaviors of the new partner toward any offspring. We also tested whether having dependent offspring on their territory attracted larger, higher-quality females, or sped up re-mating times relative to not having offspring, which could suggest that displays of paternal care or prospects of energetic benefits gained by brood cannibalism are attractive to females. A previous study on *V. moorii* examined partner loss in both males and females and found that while females continued to guard and raise offspring after the removal of their male partner, males were less successful and more variable in their responses after removal of their female partner (Zimmermann et al. 2021). Furthermore, prior research has suggested that relative to females, male investment into brood care is exceptionally low, with males preferring to protect their territory rather than the offspring on it (Zimmermann et al. 2019). Taken together, this raises doubts as to whether male and female defense behavior on the territory, which has been labeled as biparental care, is indeed equally for the benefit of the offspring. For this reason, we only focused on male re-mating strategies in this study.

## METHODS

### Study species

Parental care in *V. moorii* lasts for approximately 100 days (Rossiter 1991), and consists largely of maintaining egg hygiene and defending the brood from predators (Smith and Wootton 1995; Balshine and Sloman 2011; Zimmermann et al. 2019). Both parents defend against conspecific and heterospecific territory intruders, some of which pose threats to the offspring and some of which compete for the territory itself (Karino 1997; Sturmbauer, Fuchs, et al. 2008; Ota et al. 2012; Zimmermann et al. 2019). Natural rates of territory takeovers, however, are currently unknown. Intruders that pose a predation threat to the parents are generally rare in the littoral habitat (e.g., *Boulengerochromis microlepis*, *Lates* sp., pers. obs. by the authors). Genetic parentage analyses have revealed that extra-pair paternity occurs at very high rates, and on average 25–50% of offspring are sired by extra-pair males, which has likely led to dampened paternal investment in this system relative to maternal investment (Sefc et al. 2008; Bose et al. 2018; Zimmermann et al. 2019). Previous studies have not detected any clear relationship between brood paternity and male care behaviors (Zimmermann et al. 2019, 2021). Several mechanisms can potentially lead to partner loss in *V. moorii*, including partner desertion, predation, or coastal fishing pressure. While natural rates of partner desertion are unknown, they are likely to be low because partner desertion would also entail the loss of a territory, which is highly limiting (Sturmbauer, Hahn, et al. 2008).

### Experimental setup

We conducted this field experiment from September 4th until October 6th 2018 at the eastern shore of Mutondwe Island, Lake Tanganyika, Zambia (8°42ʹ29.4″S 31°07ʹ18.0″E). This site consists of rocky habitat ranging down to a depth of 16 m and contains a large population of breeding *Variabilichromis moorii*. While on SCUBA, we identified pairs of males and females that were defending territories and haphazardly chose 42 territories that contained dependent offspring (ranging between 2–6 m in depth). At each territory, we caught the male and female by using gill nets (0.5 cm mesh width), sexed them according to the morphology of their genital papillae, and measured them for total length to the nearest mm. We marked the male parents for future identification as the territory-owning individuals. This was done by clipping the dorsal edge of each male’s caudal fin, and also by clipping the second or third dorsal fin ray, which permitted longer-term re-identification. We collected the full brood from each territory by gently and manually maneuvering them into a transparent plastic bag following methods detailed in Zimmermann et al. (2021). We then took pictures in triplicate of the brood in the bag against a scaled white background under water. We later used these pictures to quantify brood sizes and to measure offspring sizes to the nearest mm. Brood size was taken to be the highest offspring count for each brood based on the three pictures. Offspring size was quantified in ImageJ (v1.53t) by taking the average of four fry oriented so their lengths could be measured against the scale.

After manipulation, we assigned each territory to one of two treatments and did so haphazardly with respect to male and territory traits. In one treatment (*n* = 23 territories), the males and their offspring were returned to their territories, while in the other treatment (*n* = 19 territories), the males were returned without their offspring. As previous studies have detected no clear relationship between brood paternity and paternal care (Zimmermann et al. 2019, 2021), we abstained from subsampling the broods for genetic paternity testing to keep brood sizes stable. Females were removed in both treatments and held in concrete ponds at a nearby facility for the duration of the experiment; some of them were also used in other studies (e.g., Bose et al. 2020). We also aimed to balance offspring size between the two treatment groups, and did so based on visual approximations of the offspring during their capture while on SCUBA. The average body size of the offspring that were returned to their territories did not differ significantly from that of the offspring removed from their territories (mean ± SD, “brood returned”: *n* = 23 territories, 10.6 ± 5.8 mm, “brood removed”: *n* = 19 territories, 9.5 ± 4.4 mm; Wilcoxon rank sum test, *W* = 232, *P* = 0.75). Males that were returned to their territories with their broods also did not differ significantly in average body size from the males that were returned to their territories without their broods (mean ± SD for average size of territory owners, “brood returned”: *n* = 23 males, 86.5 ± 4.6 mm, “brood removed”: *n* = 19 males, 85.5 ± 5.7 mm; *t*-test, *t*_*34.4*_ = 0.60, *P* = 0.55). However, males in the “brood returned” group initially had larger broods on average than males in the “brood removed” group (mean ± SD, “brood returned”: *n* = 23 territories, 47.4 ± 36.9 offspring, “brood removed”: *n* = 19 territories, 26.1 ± 23.7 offspring; Wilcoxon rank sum test, *W* = 311, *P* = 0.02, note that average brood size in this population is 31.9 ± 19.6, Zimmermann et al. 2022). This was done to minimize the number of offspring that we euthanized in this study. Furthermore, the males and territories in the “brood removed” group did not visually differ from those in the “brood returned” group in any other respects (e.g., location in the study quadrat, water depth, algal cover, etc.), which would suggest that they differed in quality or condition. The manipulation thus produced a comparison between males with no offspring and males with offspring. Removed offspring were euthanized in an overdosed bath of MS-222 (1 mg/liter lake water) immediately after diving, and both offspring and parental fin clips were stored in 99.9% ethanol for use in other studies (e.g., Zimmermann et al. 2021).

### Behavioral scoring and post-manipulation territory checks

In order to observe how single males interacted with new potential mates after partner vacancies were created, we set up cameras by each male’s territory (using GoPro Hero 5 session cameras), and recorded video of the male’s behavior immediately upon returning him to his territory. These videos also captured heterospecifics as they intruded on territory space and were repelled by the residents (following Zimmermann et al. 2019, 2021). These videos lasted approximately 2 h. We also returned to the territories to perform a visual check at 2 h post manipulation (2 HPM) for the presence of the original male, his brood (in the treatment where brood was returned to the territory), and any newly arriving fish that were interacting with the male or taking up residency on the territory. We performed another check after 24 h (24 HPM), and then haphazardly returned to these territories for additional follow-up checks an additional one or two times between 2- and 31-day post manipulation. To minimize disturbance at the territories, we did not recapture the males or their offspring at the subsequent checks but rather recorded presence/absence. Preliminary data suggested that once a vacancy is created at a territory, particularly when a female breeding position becomes available, new individuals prospect at these territories very quickly, typically within minutes to hours (personal observations made by the authors). Whenever a new fish was detected at the territory during the 2 HPM or 24 HPM check, we caught the newcomer, sexed and measured them for total length (to the nearest mm), and clipped their caudal fin as well as a dorsal fin ray for future re-identification. Here, if the new fish was a female, we clipped the ventral edge of her caudal fin to differentiate her from the male, who had been clipped on the dorsal edge of their caudal fin (see above).

One researcher scored the videos from the first 2 h after the male had been returned to his territory, and observed the arrivals of new conspecifics to the territory. Many new conspecifics were present at the territory only transiently; they did not interact with the male or were even chased away by the male, and so we did not score these fish. However, there was sometimes one newcomer that was allowed to remain on the territory. They would often engage in affiliative behaviors with the male and begin defending the territory from intruders themselves. We chose to start scoring behavior at the appearance of this individual. We recorded all social interactions between the territorial male and the newcomer. In particular, we quantified aggressive and affiliative behaviors performed between the pair (see Table 1 for detailed description of behaviors) and we recorded whether the newcomer preyed on the offspring (in the “brood returned” treatment). Both the resident males and newcomers also engaged in defense behaviors against intruding conspecifics and heterospecifics and so we scored these separately. Behavioral scoring began at the first arrival of the newcomer and continued for either 30 min or until the new fish disappeared from the screen for more than 60 s. We did this because it was not possible to verify with certainty the identity of a new, unclipped, conspecific if they left the field of view for a prolonged period of time before returning. However, in most cases where a new conspecific was visible in the videos interacting with the male and exhibiting territorial defense, a new conspecific was also present on the territory at the 2 HPM mark (this occurred in 19 out of 26 video trials). This suggests that the new fish that we captured and fin clipped at the 2 HPM mark were the same newcomers that we behaviorally scored from the videos. In a few instances, the new fish could not be captured at the 2 HPM check and so we ascertained their sex based on the video footage instead (four fish in total, see * and **footnotes in Table 2); males and females can be visibly distinguished from a close-up, side-on view showing their genital papillae.

**Table 1:** Ethogram of *Variabilichromis moorii* behaviors scored from videos that capture the first 2 h after our experimental manipulations

Behavior	Category	Description
Attack	Aggressive	The fish darts toward an intruder in order to chase or bite the opponent.
Rush	Affiliative	Quick movement toward the other fish but with a sudden stop before contact. Often followed by display or soft touch behavior, and never followed by an attack.
Soft touch	Affiliative	Focal fish gently nudges other fish. Never followed by biting or chasing. Can be repeated several times or reciprocally performed by both fish.
Display	Affiliative	Presenting the lateral side with erected fins. Body posture is not rigid as is typical for aggressive displays of other cichlids.
Head-up display	Submissive	Presenting the lateral side with folded fins while the head points upwards. Often accompanied with tilting the body to show the belly toward other fish.

The social interactions between the male territory owners and the newly arriving females could involve any of the above behaviors. However, when the males and females interacted with intruding con- and heterospecifics, the interactions were always aggressive.

**Table 2:** Breakdown of sample sizes between treatments at the different time points of this field experiment

		Treatment	
		Brood returned	Brood removed
**Setup**	Num. territories manipulated in experiment	23	19
	Average male size (mm ± SD)	86.5 ± 4.6	85.5 ± 5.7
	Average brood size (num. offspring ± SD)	47.4 ± 36.9	NA
**Video** **(2 h)**	Num. territories where a new conspecific arrived on video and behavior was scored	13	13
**2 HPM** **check**	Num. territories checked at 2 HPM	23	19
	Num. territories with a new female present	13	12^*^
	Num. new females measured and marked^****^	13	9^**^
	Num. territories with offspring still present	15	NA
**24 HPM check**	Num. territories checked at 24 HPM	22^***^	17^***^
	Num. territories with a new female present	18	14
	Num. females with measures and markings	18	11
	Num. territories with offspring still present	3	NA

^*^One newcomer seen on the video was not present at the territory at the 2 HPM check, but its sex (female) was determined from side-on views during the video (see Methods)

^**^Three newcomers evaded capture at the 2 HPM check and so could not be measured or marked by fin clipping. Since we did not attempt to catch them again, to minimize disturbance, these fish were also not measured or marked at the 24 HPM check. However, their sex (female) was determined from side-on views during their videos (see Methods)

^***^Note that three territories that were checked at 2 HPM could not be checked again at 24 HPM due to logistical challenges.

^****^Note that two additional females (one in each treatment) had to be excluded from the analysis examining the probability that new females seen at 2 HPM would still be present on the territories at 24 HPM. This is because, although they were captured, they escaped fin clipping at the 2 HPM check.

### Statistical analysis

All statistical analyses were performed in R 4.1.2 (R Core Team 2021). We first tested whether the probability of a new female arriving at the territory within the first 2 HPM was influenced by the presence of offspring. We fit a generalized linear mixed-effects model (GLMM, *n* = 19 “brood removed” and 23 “brood returned” territories, see Table 2) with a binomial error distribution (using the R package glmmTMB, Brooks et al. 2017). We included the presence of a new fish at the 2 HPM check as a binary response variable, and included “treatment” (“brood returned” vs “brood removed”) as a categorical predictor variable and “male size” (in mm) as a continuous covariate. Our manipulations of territories took place over 12 different days (several territories were manipulated each day), and so we included “date of manipulation” as a random intercept to account for the non-independence of data from the same days.

We used an analysis of covariance (ANCOVA) to test if having offspring on the territory attracts differently sized females to the territory. To maximize our sample size, this model combined data from territories in which new females could be measured during the 2 HPM and 24 HPM checks (*n* = 18 territories in the “brood returned” treatment and 11 territories in the “brood removed” treatment, see Table 2). We used the sizes of the new females as the response variable and included “treatment” as a predictor variable along with “male size” as a covariate.

Next, we tested whether the presence of offspring affected the probability that new females would remain on the territory. Here, we used all territories where a new female was present at the 2 HPM check and fit a GLMM with binomial error distribution. We included a binary, yes-no, response variable indicating whether the female that was observed at the 2 HPM check was still located at the same territory at the 24 HPM check (with “yes” meaning that the same female was still present and “no” meaning that she had disappeared, possibly having been replaced by a new female) and “treatment” as a predictor variable. We also included “male size” as a covariate and “date of manipulation” as a random intercept. Note that while *n* = 25 territories had females present at the 2 HPM check, five of these territories were omitted from this analysis. This is because logistical challenges stopped us from checking them again at 24 HPM (*n* = 3 territories, see ***footnote in Table 2), or the females were captured at 2 HPM for measurement, but escaped and returned to the territory before they could be fin clipped, thereby hampering our ability to re-identify them at a later check (*n* = 2, see ****footnote in Table 2). To minimize (and standardize) handling, we only attempted to capture each fish once throughout the experiment.

We also examined whether the early behavioral interactions between the territorial males and the new females, that is, those behaviors that we scored during the observation periods within the videos documenting the first 2 HPM, were influenced by the presence or absence of offspring. We fit a GLMM (*n* = 13 “brood removed” and 13 “brood returned” territories, see Table 2) assuming a negative binomial error distribution (using the nbinom1 family in glmmTMB, Brooks et al. 2017), and included the counts of attack behaviors by the males and new females toward one another as the response variable. We included “treatment,” “sex,” and the relative size difference between the male and female [calculated as (male size - female size)/ (male size + female size)]) as predictor variables. We also included “territory ID” as a random intercept to accommodate the non-independence of behavioral data coming from the male and female on each territory. Additionally, we set “observation duration” (seconds, log-transformed) as a model offset to account for the different time windows when behavior was scored. We also fit a second GLMM, identical to the first, but we included counts of all affiliative behaviors between the pair as the response variable rather than aggressive behaviors.

Next, we examined whether new females would adopt territorial behaviors upon their arrival at a territory, and whether such territorial behavior was influenced by the presence or absence of offspring. Using behavior scored from the videos documenting the first 2 HPM, we fit a GLMM (*n* = 13 “brood removed” and 13 “brood returned” territories, see Table 2) with a beta-binomial error distribution to account for over-dispersion. We included defense share as the response variable, which was calculated as the female proportion of territory defense behaviors performed against each of two intruder types, con- and heterospecifics. We included “treatment,” and “intruder type” (conspecific, heterospecific) as predictor variables. We considered the interaction between “treatment” and “intruder type” but dropped the interaction from the final model because it did not significantly improve model fit based on a likelihood ratio test using the “lrtest” function from R package lmtest (Zeileis and Hothorn 2002, χ^2^ = 1.87, *P* = 0.17). We included “territory ID” as a random intercept. Finally, we also tested whether males and females ever deviated from an egalitarian division of territory defense by inspecting the model intercept term. We did this for each combination of treatment and intruder type by changing the reference level for each factor (using the “relevel” function in R) and refitting the model.

Lastly, we tested whether the presence of a new female negatively affected the survival of any offspring that were present on a territory. Here, we analyzed only territories from the “brood returned” treatment group at the 2 HPM check (*n* = 23 territories, see Table 2). The model included a binary response variable indicating whether offspring were still present on the territory. The model also included “new female presence” (yes/no) and “male size” as predictor variables. We additionally included “brood size” as a predictor variable in this model because it may have taken new females more time to consume larger broods compared to smaller broods. We fit a GLM using Firth´s bias-reduced logistic regression (R package logistf, Ploner et al. 2010), because there was zero variance in one of our experimental groups; that is, all broods were still present on territories where no female arrived within the first 2 h of the experiment.

### Ethics

This study was carried out with the approval of the ethics committee of the University of Graz (permit number 39/50/63 ex 2018/19). The field experiment was conducted with the permission of the Fisheries Department of Zambia and under study permits issued by the government of Zambia (SP 007216, SP 008735). The study species is listed as “Least Concern” under the name of *Neolamprologus moorii* in the IUCN Red List of Threatened Species 2006. The procedures used in this study were in line with the guidelines set by the Animal Behavior Society (Animal Behaviour, 135: I- X, 2018) regarding the treatment of animals in research and teaching. Only trained personnel handled the fish.

## RESULTS

### Social partner vacancies are quickly filled by new females

No males abandoned their territories across the first 24 HPM. The single males quickly received interactions from new conspecifics, and in all cases where a newcomer arrived and remained on the territory, it was a female. Out of the 42 males in this experiment, 25 (~60%) had been joined by a new female *V. moorii* at the 2 HPM mark (Table 2); a new female was present at the 2 HPM check in 13 of the 23 territories with offspring, and in 12 of the 19 territories without offspring. After 24 h, 39 territories were re-checked and 32 of them (~82%) had a female present alongside the male (14 out of 17 territories in the “brood removed” group and 18 out of 22 territories in the “brood returned” group, Table 2).

There was no significant difference between the treatment groups in the chances of attracting a new female within the first 2 HPM (GLMM, *n* = 42, *z* = 0.46, *P* = 0.65). The size of the males did not significantly correlate with their likelihood of having been joined by a female at the 2 HPM check (GLMM, *n* = 42, *z* = 0.26, *P* = 0.79).

The size of the single males was positively related to the size of their new females (ANCOVA, *F*_1,26_ = 4.5, *P* = 0.04), but the presence of offspring on the territory did not correlate with the sizes of the females (ANCOVA, *F*_1,26_ = 0.08, *P* = 0.78).

Of the new females that were fin clipped at the 2 HPM check, 70% of them (14 out of 20; two females had to be excluded, see ****footnote in Table 2) were still present on territories that we also checked at 24 HPM. There was neither an effect of treatment (GLMM, *n* = 20, *z* = −0.28, *P* = 0.78) nor of male size (GLMM, *n* = 20, *z* = 0.12, *P* = 0.90) on the likelihood that the new female would stay until the 24 HPM mark.

### New females engage in more social behavior with male territory-owners than vice versa

Overt aggression between the males and females was rare during our observations. Half of the newly formed pairs showed no aggression toward each other at all (median + IQR; female aggression: 0 + 0.1 attacks per minute, range = 0–0.5 attacks/min; male aggression: 0 + 0 attacks/min, range = 0–0.2 attacks/min). We detected no significant relationship between the presence of offspring on the territory and the aggression displayed between the males and the females (GLMM, *n* = 46, treatment, *z* = 0.68, *P* = 0.50), however, males performed significantly fewer attacks against the new female than vice versa (GLMM, *n* = 46, sex, *z* = −2.50, *P* = 0.012). The size difference between the males and the new females also did not correlate with the number of aggressive behaviors performed between the two (GLMM, *n* = 46, relative size difference, *z* = 0.57, *P* = 0.57).

**Figure 1:**
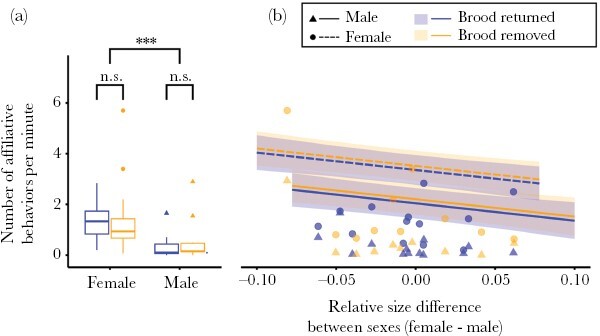
(a) Females performed more affiliative behaviors toward males than males did toward females, irrespective of treatment. (b) The number of affiliative behaviors by females toward males tended to decreased as females became larger compared to the males. Box plots in (a) show medians (thick horizontal lines), first and third quartiles (boxes) and the range of data within 1.5 interquartile distances above and below the interquartile (whiskers). Panel (b) presents raw data points as well as model predicted fits and standard error. Non-significant results are indicated by n.s., while significant results at *P* < 0.001 are indicated by ***.

When examining the frequencies of affiliative behaviors, we found that males, on average, engaged in fewer affiliative behaviors toward the females than vice versa (median + IQR; female affiliation: 1.2 + 1.0 behaviors per minute, range = 0.1–5.7 behaviors/min; male affiliation: 0.1 + 0.4 behaviors/min, range = 0–2.9 behaviors/min; GLMM, *n* = 46, sex, *z* = −7.37, *P* < 0.0001, Figure 1a). The presence of offspring on the territory did not significantly affect the expression of affiliation between the male and the female (GLMM, *n* = 46, treatment, *z* = 0.57, *P* = 0.57). Finally, though not statistically significant, there was a trend for the size difference between the females and the males affected the number of affiliative behaviors insofar as females performed fewer affiliative behaviors toward the males as they increased in size relative to the males (GLMM, *n* = 46, relative size difference, *z* = −1.73, *P* = 0.08, Figure 1b).

### New females prioritize defense against conspecifics

**Figure 2:**
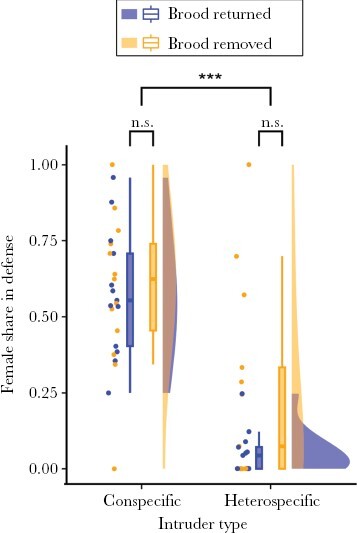
Raincloud plots showing female share in territory defense against intruding con- and heterospecifics. Female defense share against con- and heterospecifics did not differ significantly with treatment. However, female defense share against heterospecifics was significantly lower than against conspecifics. Box plots show medians (thick horizontal lines), first and third quartiles (boxes) and the range of data within 1.5 interquartile distances above and below the interquartile (whiskers). Dots (left of the box plots) and density plots (right of the box plots) show the distribution of observed defense shares. Non-significant results are indicated by n.s., while significant results at *P* < 0.001 are indicated by ***.

Most females joined the males in territorial defense and were similarly involved in defense compared to the males (defense behaviors, median + IQR, for males: 1.5 + 0.8 defense behaviors/minute; for females: 0.8 + 1.1 defense behaviors/minute; paired Wilcoxon Signed Rank test, *n*_males_ = *n*_females_ = 26 individuals, *V* = 227.5, *P* = 0.19). Females were more likely to engage in territorial defense against conspecifics encroaching on the territory than against heterospecifics (GLMM, *n* = 52, intruder type, *z* = −7.33, *P* < 0.0001, Figure 2, Table 3a). On average, 63 ± 25% (mean ± SD, *n* = 26 pairs) of the defense behaviors performed by the male-female pair were directed at intruding conspecifics, and of this, 58 ± 23% of these behaviors were performed by the females (compared to only 15 ± 25% that the females participated in against heterospecifics). The presence or absence of offspring on the territory did not significantly affect the amount of defense behaviors expressed by the females relative to the males (GLMM, *n* = 52, treatment, *z* = 1.09, *P* = 0.27, Figure 2, Table 3a). While males and females did not deviate from egalitarian defense when faced with conspecific intrusions in the “brood returned” treatment (GLMM, *n* = 52, intercept, *z* = 1.18, *P* = 0.24, Table 3a), females participated in significantly less defense than males when faced with heterospecific intrusions regardless of treatment (Table 3b,c). Females also defended significantly more than males against conspecific intruders in the “brood removed” treatment (Table 3d).

**Table 3:** (a) Effects of treatment and intruder type on female defense share (based on GLMM with beta-binomial error structure). Intercept tests for egalitarian division of defense between males and females when predictor variables are set to their reference levels. (b)–(d) Model intercept terms for releveled models, each with a different combination of predictor variable reference levels.

		Estimate ± SE	*z*	*P*
(a)	**Main model** reference level for treatment is “brood returned” reference level for intruder type is “conspecific”			
	Intercept	0.27 ± 0.23	1.18	0.24
	Treatment	0.33 ± 0.30	1.09	0.27
	Intruder type	−2.47 ± 0.34	−7.33	**< 0.0001**
(b)	**Releveled model** reference level for treatment is “brood returned” reference level for intruder type is “heterospecific”			
	Intercept	−2.21 ± 0.30	−7.31	**< 0.0001**
(c)	**Releveled model** reference level for treatment is “brood removed” reference level for intruder type is “heterospecific”			
	Intercept	−1.88 ± 0.33	−5.65	**< 0.0001**
(d)	**Releveled model** reference level for treatment is “brood removed” reference level for intruder type is “conspecific”			
	Intercept	0.60 ± 0.25	2.42	**0.016**

Significant results as α = 0.05 are in bold.

### New females were highly cannibalistic toward any present offspring

**Figure 3:**
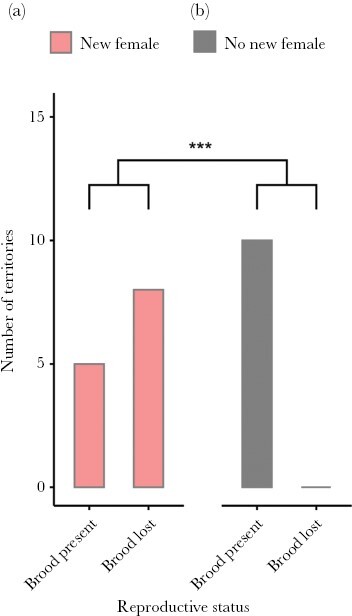
Variabilichromis moorii territories receiving the “brood returned” treatment (*n* = 23) and subsequently checked 2 h post manipulation (HPM). (a) When a new female arrived at a territory within 2 HPM, there was an acute loss of the brood in an appreciable number of cases. (b) However, in cases where no new female had arrived at a territory by 2 HPM, the broods that had been returned to the males were all still present. Significant results at *P* < 0.001 are indicated by ***.

Of the 23 territories that had their brood returned, eight were found without offspring at the 2 HPM check. In each of these cases, a female had also arrived to the territory. The remaining 15 territories still had offspring present at the 2 HPM check, and in five of these cases a female had also arrived. All newly arriving females were seen to prey on the offspring present on the territories during the video scoring (*n* = 13 territories of the “brood returned” treatment), and brood cannibalism by the newcomers was also confirmed during the in-person check of the territories. While the females were highly infanticidal, the males did not clearly attempt to prevent this infanticide, neither during the videos nor at our territory checks. Whether or not offspring survived on the territories up to the 2 HPM mark was significantly and negatively associated with the presence of a new female (GLM, female presence, *n* = 23, χ^2^ = 11.58, *P* = 0.0007, Figure 3). Furthermore, offspring were more likely to still be alive when they were on a large male’s territory relative to a small male’s territory (GLM, male size, *n* = 23, χ^2^ = 4.61, *P* = 0.032). Larger broods did not withstand the infanticidal attempts by females longer than small broods (GLM, brood size, *n* = 23, *X*^2^ = 0.78, *P* = 0.38). Out of the 15 territories that still had offspring at the 2 HPM check (see Table 2), 12 of them had new females by the time of the 24 HPM check, but only three of the 15 territories still retained their broods (two with a female present and one without a female).

## DISCUSSION

In this field experiment, we examined the process of male re-mating in the socially monogamous cichlid species *Variabilichromis moorii*, by experimentally separating males from their female partners and then either removing or not removing their dependent offspring. None of the single males abandoned after our experimental manipulations, likely because abandonment would have entailed also losing their territory. We monitored the males as they subsequently interacted with potential new mates either in the presence of absence of their broods from their previous partner. New females prospected very quickly at the territories of the single males, sometimes within minutes of the vacancy being created, and this was irrespective of the presence of a brood of offspring. New females establishing residency at a territory engaged in more affiliative behaviors toward the resident males than vice versa, and also prioritized fending off conspecifics, rather than heterospecifics, that were approaching the territory. Overall, aggression between the resident males and the new females was very low, even when new females were infanticidal toward offspring that were still present from a previous partnership.

The consequences of partner loss in biparental systems are often studied from the perspective of mate desertion. Mate desertion during brood care occurs when the abandoner can expect future reproductive payoffs for deserting that outweigh those for continuing to assist with biparental care. Such desertion is facilitated when the remaining parent is also willing, or forced, to care for the offspring (Trivers 1972; Dawkins and Carlisle 1976; Székely et al. 1996), though the single parent may do so with an adjusted level of parental investment (Beissinger and Snyder 1987; Harrison et al. 2009; Lehtonen et al. 2011). However, partner loss can also occur when one parent dies, leaving the other with sole custody of the brood (e.g., Santema and Kempenaers 2018). Parental responses to partner loss should not be expected to be similar in systems where partner loss occurs primarily through mortality compared to systems where it occurs primarily through mate desertion (Székely et al. 1996). While natural mate desertion rates are unknown in *V. moorii*, there are a number of lines of evidence to suggest that it is rare. First, mate desertion would also involve territory desertion, and would therefore be highly costly because territory competition is high in *V. moorii* (Karino 1998; Sturmbauer, Hahn, et al. 2008; Ota et al. 2012). Second, although recapturing individuals across years is rare, our extensive microsatellite genotyping of this study population across numerous successive breeding seasons has found no evidence of regular mate desertion followed by re-pairing with alternative mates.

Previous research in *V. moorii* has shown that brood-tending females can still care for offspring in the absence of their male social partners, but males will lose their offspring within several days after becoming single (based on observations in Zimmermann et al. 2021). Single females tend not to be joined by new males while they continue to care (Zimmermann et al. 2021), despite there existing a large pool of reproductively mature floater males in the population to re-pair with (Bose et al. 2018). This suggests that single females prefer to delay re-mating. In the present study, we show that single males are often joined by new females very quickly, sometimes within minutes of a vacancy being created. Having offspring on a territory did not attract larger females, or reduce a male’s time until re-pairing, suggesting that offspring presence, or displays of male care, may be relatively inconsequential for mate choice in this species. Males also aggressed very little toward the new females despite the risk they posed to their offspring. Together, this suggests that the sexes differ in their re-mating strategies. While single females appear to prioritize offspring care (Zimmermann et al. 2021), single males appear to prioritize re-mating over raising their current brood.

New females arriving at a territory received very little aggression from the resident males, and this level of aggression did not depend on whether the males still had offspring from their previous partnerships. New females were also more affiliative toward the resident males than the males were toward the females, particularly when the new females were smaller in body size, suggesting that affiliation might be a tactic used by joiners when their resource holding potential does not allow them to join by aggressive takeover. Our observations that joining females were affiliative with the males while also being defensive toward intruding conspecifics, aligns with the notion that our experimental vacancies generated competition among females seeking a territory (Ahnesjö et al. 2001). The apparent abundance of potential female mates may also be expected to promote choosiness in males (Edward and Chapman 2011), however a more targeted study would be required to evaluate this possibility.

If the single males still had offspring present from their previous partnerships, then the new females attempted to cannibalize the offspring, sometimes immediately after their arrival. In fact, offspring survival was strongly and negatively correlated with the arrival of new females. Interestingly, despite the ongoing infanticide, the resident males did not express increased aggression toward the females, which would have suggested paternal brood defense. This is despite the males being capable of fighting and fending off individuals substantially larger than themselves (H.Z., A.B. personal observations). We therefore interpret the passivity by the males to be a tactic within the male re-mating strategy. This apparent lack of response is in contrast to other species where the original caregiver typically attempts to resist threats to their offspring (e.g., strong maternal aggression as seen in lions, Packer et al. 1990; collared lemmings, Mallory and Brooks 1978; *Neolamprologus pulcher*, Jindal et al. 2017). Brood size, age, and paternity are all relevant factors determining the reproductive value of offspring (Alonzo and Klug 2012; Bose 2022) and may explain our experimental males’ lackadaisical behavior in the face of infanticide. In this experiment, we avoided territories with very large broods to reduce the impacts that our manipulations would have on the population. We also focused on relatively young broods, as offspring become progressively more difficult to capture with age. Although we did not assess brood paternity in this experiment, males of this species typically sire only 50 – 75% of the offspring in their broods (Sefc et al. 2008; Bose et al. 2018; Zimmermann et al. 2019, 2022), and previous work has shown that paternal behaviors do not correlate clearly with brood paternity (Zimmermann et al. 2019). We conducted *post-hoc* analyses to test whether brood survival (i.e., brood presence/absence at 2 HPM) and male aggression toward the new females were significantly correlated with brood size or brood age (using offspring size as a proxy for age). Neither brood size nor age in our sample were significantly related to brood survival or male aggression (see Supplementary Tables S1 and S2). Future research will be needed to characterize the responses of both males and their new mates when broods are larger and older (i.e., of higher putative reproductive value to the males). An intriguing possibility is that the brood itself could represent a nuptial gift to the new females to improve their future reproductive prospects (Lewis and South 2012). However, in this study, we could not detect convincing subsequent benefits for males that had offspring to offer to new females. Out of our experimental territories, we conducted follow-up checks on 15 of them between 2 and 5 weeks post manipulation. Ten original males were still present on these territories, and seven of them (four males from the “brood removed” treatment and three males from the “brood returned” treatment) were still paired with the same female that had arrived soon after our manipulations, and only two had produced a new brood yet (one “brood removed” territory with freshly hatched offspring 26 days after manipulation and one “brood returned” territory with new eggs 15 days after manipulation).

Overall, our results are consistent with previous research, which has suggested that males show low paternal investment into their broods (Zimmermann et al. 2019, 2021). In a previous study (Zimmermann et al. 2019), maternal females undertook most of the defense against heterospecific intruders that were egg or offspring predators, whereas defense against species that compete for territory space (including conspecifics and certain heterospecifics) was shared between paternal males and maternal females. This suggests that defense behavior by maternal females aligns well with offspring protection, whereas defense behavior by paternal males aligns more with territory protection (Zimmermann et al. 2019). However, since the new females in our current study had no direct fitness interests in the broods, they, consistently, tended to ignore heterospecifics (including potential egg and offspring predators), and instead focused on fending away conspecifics. We interpret this focus on conspecific defense by new females as an effort to retain their newly won paired status. Thus, males transitioning from their old partner to a new partner are presumed to shift their workload to accommodate this new lack in heterospecific defense. Such behavioral flexibility mirrors changes that are predicted to occur when pairs use negotiation strategies to coordinate cooperative behavior (e.g., parental negotiation strategies, Smiseth 2019). One intriguing way of viewing our manipulation is through the lens of a partner handicapping experiment. Here, the female partner’s investment into heterospecific defense was experimentally reduced (handicapped, by replacing the male’s partner with one that is focused primarily on conspecific defense). In response, males flexibly increased their investment into heterospecific defense, though it is likely that their defense was still motivated by territory retention more so than offspring protection. Thus, our work shows that males have the capability of flexibly compensating for changes in their partner’s defense behavior at least in the short term.

In this experiment, we showed that *V. moorii* males suddenly facing uniparental care (due to partner loss) will submit to forfeiting their current brood for quick re-mating opportunities. New females were quick to fill the partner vacancies that we created, and the males did not take any measures to deter females from cannibalizing their offspring. The new females defended the territory from intruders, but focused their defense against conspecifics, which we interpret as an effort to repel rival females. Overall, the presence of offspring from a previous partnership had no detectable effects on the males’ behavior, which is in line with previous studies suggesting that males of this species have very low parental investment into their offspring (Zimmermann et al. 2019, 2021). Drawing comparisons to earlier experiments in which partner vacancies left single mothers caring for offspring alone (Zimmermann et al. 2021), we emphasize how re-mating strategies can differ between the sexes with one parent willing to continue to caring for offspring alone, but the other willing to sacrifice their offspring to re-mate quickly.

## Supplementary Material

arad045_suppl_Supplementary_Material

## Data Availability

Analyses reported in this article can be reproduced using the data provided by Zimmermann et al. (2023).

## References

[CIT0001] Ahnesjö I , Kvarnemo C, Merilaita S. 2001. Using potential reproductive rates to predict mating competition among individuals qualified to mate. Behav Ecol. 12(4):397–401.

[CIT0002] Alonzo SH , Klug H. 2012. Paternity, maternity, and parental care. In: Royle NJ, Smiseth PT, Kölliker M, editors. The evolution of parental care. Oxford: Oxford University Press. p. 189–205.

[CIT0003] Balshine S , Sloman KA. 2011. Parental care in fishes. In: Farrell AP, editor. Encyclopedia of fish physiology: from genome to environment. Vol. 1. San Diego: Academic Press. p. 670–677.

[CIT0004] Beissinger SR , Snyder NFR. 1987. Mate desertion in the snail kite. Anim Behav. 35(2):477–487.

[CIT0005] Bose APH. 2022. Parent–offspring cannibalism throughout the animal kingdom: a review of adaptive hypotheses. Biol Rev. 97(5):1868–1885. 35748275 10.1111/brv.12868

[CIT0006] Bose APH , Zimmermann H, Henshaw JM, Fritzsche K, Sefc KM. 2018. Brood-tending males in a biparental fish suffer high paternity losses but rarely cuckold. Mol Ecol. 27(21):4309–4321. 30182504 10.1111/mec.14857 PMC6221093

[CIT0007] Bose APH , Zimmermann H, Winkler G, Kaufmann A, Strohmeier T, Koblmüller S, Sefc KM. 2020. Congruent geographic variation in saccular otolith shape across multiple species of African cichlids. Sci Rep. 10(1):12820. 32733082 10.1038/s41598-020-69701-9 PMC7393159

[CIT0008] Brooks ME , Kristensen K, van Benthem KJ, Magnusson A, Berg CW, Nielsen A, Skaug HJ, Maechler M, Bolker BM. 2017. {glmmTMB} balances speed and flexibility among packages for zero-inflated generalized linear mixed modeling. R J. 9(2):378–400.

[CIT0010] Dawkins R , Carlisle TR. 1976. Parental investment, mate desertion and a fallacy. Nature. 262(5564):131–133.

[CIT0011] Dominey WJ , Blumer LS. 1984. Cannibalism of early life stages in fishes. In: Hausfater G, Hrdy SB, editors. Infanticide: comparative and evolutionary perspectives. 1st ed. New York: Aldine. p. 43–64.

[CIT0012] Ebensperger LA. 1998. Strategies and counterstrategies to infanticide in mammals. Biol Rev. 73(3):321–346.

[CIT0013] Edward DA , Chapman T. 2011. The evolution and significance of male mate choice. Trends Ecol Evol. 26(12):647–654. 21890230 10.1016/j.tree.2011.07.012

[CIT0014] Goldberg RL , Downing PA, Griffin AS, Green JP. 2020. The costs and benefits of paternal care in fish: a meta-analysis. Proc R Soc B. 287(1935):20201759. doi:10.1098/RSPB.2020.1759. PMC7542810 32933439

[CIT0015] Harrison F , Barta Z, Cuthill I, Székely T. 2009. How is sexual conflict over parental care resolved? A meta-analysis. J Evol Biol. 22(9):1800–1812. 19583699 10.1111/j.1420-9101.2009.01792.x

[CIT0016] Hrdy SB. 1974. Male-male competition and infanticide among the Langurs *(Presbytis entellus)* of Abu, Rajasthan. Folia Primatol. 22(1):19–58. 10.1159/000155616 4215710

[CIT0017] Hrdy SB. 1979. Infanticide among animals: a review, classification, and examination of the implications for the reproductive strategies of females. Ethol Sociobiol. 1(1):13–40.

[CIT0018] Jindal S , Bose APH, O’Connor CM, Balshine S. 2017. A test of male infanticide as a reproductive tactic in a cichlid fish. R Soc Open Sci. 4(3):160891. doi:10.1098/RSOS.160891 28405376 PMC5383833

[CIT0019] Karino K. 1997. Influence of brood size and offspring size on parental investment in a biparental cichlid fish, *Neolamprologus moorii*. J Ethol. 15(1):39–43.

[CIT0020] Karino K. 1998. Depth-related differences in territory size and defense in the herbivorous cichlid, *Neolamprologus moorii*, in Lake Tanganyika. Ichthyol Res. 45(1):89–94.

[CIT0021] Lehtonen TK , Wong BBM, Svensson PA, Meyer A. 2011. Adjustment of brood care behaviour in the absence of a mate in two species of Nicaraguan crater lake cichlids. Behav Ecol Sociobiol. 65(4):613–619.

[CIT0022] Lewis SM , South A. 2012. The evolution of animal nuptial gifts. In: Brockmann JH, Roper TJ, Naguib M, Mitani JC, Simmons LW, editors. Advances in the study of behavior. Vol. 44. Cambridge, MA: Academic Press. p. 53–97.

[CIT0023] Mallory FF , Brooks RJ. 1978. Infanticide and other reproductive strategies in the collared lemming, *Dicrostonyx groenlandicus*. Nature. 273(5658):144–146. 10.1095/biolreprod22.2.192 7378528

[CIT0024] Manica A. 2002. Filial cannibalism in teleost fish. Biol Rev. 77(2):261–277. 12056749 10.1017/s1464793101005905

[CIT0025] Ota K , Hori M, Kohda M. 2012. Testes investment along a vertical depth gradient in an herbivorous fish. Ethology. 118(7):683–693.

[CIT0026] Packer C , Scheel D, Pusey AE. 1990. Why lions form groups: food is not enough. Am Nat. 136(1):1–19.

[CIT0028] Palombit RA. 2015. Infanticide as sexual conflict: coevolution of male strategies and female counterstrategies. Cold Spring Harb Perspect Biol. 7(6):a017640. doi:10.1101/CSHPERSPECT.A017640 25986557 PMC4448612

[CIT0029] Ploner M , Dunkler D, Southworth H, Heinze G. 2010. logistf: Firth’s bias reduced logistic regression. R package version 1.10. http://CRAN.R-project.org/package=logistf.

[CIT0030] Polis GA. 1984. Intraspecific predation and “infant killing’ among invertebrates. In: Hausfater G, Hrdy SB, editors. Infanticide: comparative and evolutionary perspectives. 1st ed. New York: Aldine.

[CIT0031] Pusey AE , Packer C. 2016. Infanticide in lions: consequences and counterstrategies. In: Parmigiani S, vom Saal FS, editors. Infanticide and parental care. London & New York: Routledge. p. 277–300.

[CIT0032] R Core Team. 2021. R: a language and environment for statistical computing. Vienna, Austria: R Foundation for Statistical Computing. https://www.R-project.org/.

[CIT0033] Rohwer S , Herron JC, Daly M. 1999. Stepparental behavior as mating effort in birds and other animals. Evol Hum Behav. 20(6):367–390.

[CIT0034] Rossiter A. 1991. Lunar spawning synchroneity in a freshwater fish. Naturwissenschaften. 78(4):182–184.

[CIT0036] Santema P , Kempenaers B. 2018. Complete brood failure in an altricial bird is almost always associated with the sudden and permanent disappearance of a parent. J Anim Ecol. 87(5):1239–1250. 29892974 10.1111/1365-2656.12848

[CIT0037] Sefc KM , Mattersdorfer K, Sturmbauer C, Koblmüller S. 2008. High frequency of multiple paternity in broods of a socially monogamous cichlid fish with biparental nest defence. Mol Ecol. 17(10):2531–2543. 18430146 10.1111/j.1365-294X.2008.03763.x

[CIT0038] Smiseth PT. 2019. Coordination, cooperation, and conflict between caring parents in burying beetles. Front Ecol Evol. 7:397. doi:10.3389/fevo.2019.00397.

[CIT0039] Smith C , Reay P. 1991. Cannibalism in teleost fish. Rev Fish Biol Fish. 1(1):41–64.

[CIT0040] Smith C , Wootton RJ. 1995. The cost of parental care in teleost fishes. Rev Fish Biol Fish. 5:7–22.

[CIT0041] Sturmbauer C , Fuchs C, Harb G, Damm E, Duftner N, Maderbacher M, Koch M, Koblmüller S. 2008. Abundance, distribution, and territory areas of rock-dwelling Lake Tanganyika cichlid fish species. Hydrobiologia. 615(1):57–68.

[CIT0042] Sturmbauer C , Hahn C, Koblmüller S, Postl L, Sinyinza D, Sefc KM. 2008. Variation of territory size and defense behavior in breeding pairs of the endemic Lake Tanganyika cichlid fish *Variabilichromis moorii*. Hydrobiologia. 615(1):49–56.

[CIT0043] Székely T , Webb JN, Houston AI, McNamara JM. 1996. An evolutionary approach to offspring desertion in birds. In: Nolan V, Ketterson ED, editors. Current ornithology. Boston, MA: Springer US. p. 271–330.

[CIT0044] Takeuchi Y , Ochi H, Kohda M, Sinyinza D, Hori M. 2010. A 20-year census of a rocky littoral fish community in Lake Tanganyika. Ecol Freshw Fish. 19(2):239–248.

[CIT0045] Trivers RL. 1972. Parental investment and sexual selection. In: Campbell B, editor. Sexual selection and the descent of man 1871-1971. Chicago, IL: Aldine. p. 136–179.

[CIT0046] Turillazzi S , Cervo R. 2016. Oophagy and infanticide in colonies of social wasps. In: Parmigiani S, vom Saal FS, editors. Infanticide and parental care. London & New York: Routledge. p. 213–236.

[CIT0047] Veiga JP. 2004. Replacement female house sparrows regularly commit infanticide: gaining time or signaling status? Behav Ecol. 15(2):219–222.

[CIT0048] Yanagisawa Y , Ochi H. 1986. Step-fathering in the anemonefish *Amphiprion clarkii*: a removal study. Anim Behav. 34(6):1769–1780.

[CIT0049] Zeileis A , Hothorn T. 2002. Diagnostic checking in regression relationships. R News 2: 7--10. R News 2:28.

[CIT0050] Zimmermann H , Bose APH, Eisner H, Henshaw JM, Ziegelbecker A, Richter F, Bračun S, Katongo C, Fritzsche K, Sefc KM. 2022. Seasonal variation in cuckoldry rates in the socially monogamous cichlid fish *Variabilichromis moorii*. Hydrobiol 2022:1–13. 10.1007/s10750-022-05042-0 PMC10261196 37325485

[CIT0051] Zimmermann H , Bose APH, Ziegelbecker A, Eisner H, Katongo C, Banda T, Richter F, Bračun S, Makasa L, Henshaw JM, et al. 2021. Is biparental defence driven by territory protection, offspring protection or both? Anim Behav. 176:43–56.

[CIT0052] Zimmermann H , Fritzsche K, Henshaw JM, Katongo C, Banda T, Makasa L, Sefc KM, Bose APH. 2019. Nest defense in the face of cuckoldry: evolutionary rather than facultative adaptation to chronic paternity loss. BMC Evol Biol. 19(1):200. 31684856 10.1186/s12862-019-1528-7 PMC6829816

[CIT0053] Zimmermann H , Sefc KM, Bose APH. 2023. Single fathers sacrifice their broods and re-mate quickly in a socially monogamous cichlid. Behav Ecol. doi:10.5061/dryad.0vt4b8h40 PMC10516675 37744163

