# Supplementary material for: Single fathers sacrifice their broods and re-mate quickly in a socially monogamous cichlid
Source: Behav Ecol. 2023 Jun 20;34(5):881–90. doi: 10.1093/beheco/arad045 (PMC10516675; doi:10.1093/beheco/arad045)
Supplement: arad045_suppl_Supplementary_Material [file arad045_suppl_supplementary_material.docx]

SUPPLEMENTARY MATERIALS

This file contains the results of the post-hoc analysis to test whether offspring age (represented by offspring size) and brood size affected brood survival (Table S1) and/or male aggression directed towards the new female (Table S2). To test the effect on brood survival, we fitted the model using logistic regression with Firth´s likelihood correction. We used ‘brood survival’ (yes/no) as response variable and ‘offspring size’ and ‘brood size’ as predictor variables (Table S1). To test for effects on male aggression, we fitted a generalized mixed effects model with negative-binomial error. We used the number of aggressive behaviors the males performed against the new female as response variable and again ‘offspring size’ and ‘brood size’ as predictor variables. Additionally, we applied the territory identification code as random intercept to account for non-independence of the behavioral counts for each male and further, we used the ‘observation duration’ as a model offset to account for differing time windows for the observations.

**Supplementary Table S1:** Effects of brood age (i.e., offspring size, mm) and brood size (i.e., number of offspring per brood) on brood survival (binary response variable) at the 2 HPM check. Data were fit with a logistic regression model applying Firth´s correction to the likelihood (R package ‘logistf’).

|  | **coefficient** | **SE(coef)** | ***χ^2^*** | ***p*-value** |
| --- | --- | --- | --- | --- |
| (Intercept) | 0.18 | 1.37 | -2.37 | 0.89 |
| Fry-size | 0.010 | 0.085 | -0.15 | 0.90 |
| Brood-size | 0.006 | 0.014 | -0.034 | 0.65 |

**Supplementary Table S2:** Effects of brood age (i.e., fry size, mm) and brood size (i.e., number of offspring per brood) on the number of aggressive behaviors males performed towards their new female partners after the females arrived at the territories (count response variable). ‘Observation duration’ (seconds, log-transformed) was added as a model offset to account for the different time windows when behavior was scored. Data were fit with a GLMM assuming a negative binomial error distribution (R package ‘glmmTMB’). Significant p-values (p < 0.05) are in bold.

|  | **Estimate** | **SE(est.)** | ***z*** | ***p*-value** |
| --- | --- | --- | --- | --- |
| **(Intercept)** | **-8.27** | **1.74** | **-4.76** | **<0.001** |
| Fry-size | -0.03 | 0.15 | -0.18 | 0.87 |
| Brood-size | 0.001 | 0.019 | 0.04 | 0.76 |
